# Tuméfaction gingivale chronique

**DOI:** 10.11604/pamj.2020.37.152.26060

**Published:** 2020-10-13

**Authors:** Marielle Cerci, Clément Riviere

**Affiliations:** 1 Paul Sabatier University, Department of Oral Surgery, Toulouse University Hospital, Toulouse, France

**Keywords:** Epulis, gencive, tuméfaction, Epulis, gum, swelling

## Abstract

Epulis is a common hyperplastic benign pseudotumor of the gums. It meets two criteria which are unanimous: topographical with the epulis affecting the neck of one or two adjacent teeth; benign, because the epulis is a tumor that does not recur after complete excision, does not produce metastases or lymph node involvement. We here report the case of a 70-year-old patient who was referred by his dentist to perform total mandibular edentation before making complete denture prosthesis and with a diagnosis of suspicious indurated lesion that had progressed for 3 years, spontaneously bleeding on contact and associated with homogeneous osteolytic lesion in relation to tooth 47. The patient had only a history of prostatic neoplasia which wasn’t associated with alcohol-tobacco poisoning and treated by surgery in 2012. Symptoms were vague; only low intermittent pain relieved with analgesic use. Vincent’s sign was negative. Initial radiological appearance was characterized by homogeneous osteolysis centered to the apex of the right second molar with radiopaque border, respecting the alveolar nerve canal, thus suggesting benign lesion. Anatomopathological examination showed inflammatory epulis. Clinical and radiological assessment was performed 15 days after surgery and showed good healing without pain or infection. Given the clinical and radiological findings, differential diagnoses included squamous cell carcinoma, metastasis from prostatic adenocarcinoma, inflammatory epulis or lymphoma.

## Image en médecine

L´épulis est une pseudotumeur bénigne hyperplasique des gencives fréquente, elle répondra à deux critères qui font l´unanimité, topographique: la localisation de l´épulis au niveau du collet d´une ou de deux dents contiguës; bénignité: l´épulis est en effet une tumeur qui ne récidive pas après exérèse complète, ne donne pas de métastases, ni d´envahissement ganglionnaire. Le cas d´un patient de 70 ans nous est rapporté. Celui-ci est adressé par son dentiste traitant pour réaliser une édentation mandibulaire totale avant confection d´une prothèse complète et avis concernant une lésion suspecte évoluant depuis 3 ans, indurée, saignant spontanément au contact et associée à une lésion ostéolytique homogène en regard de la dent 47. Le patient présente comme seul antécédent notable une néoplasie prostatique traitée par chirurgie seule en 2012, sans intoxication alcoolo-tabagique. La symptomatologie est fruste avec seulement de faibles douleurs intermittentes calmées par la prise d´antalgiques. Le signe de Vincent est négatif. L´aspect radiologique initial avec une ostéolyse d´aspect homogène centrée sur l´apex dentaire de la deuxième molaire droite avec un liseré radio-opaque et respectant le canal du nerf alvéolaire nous confortait dans l´hypothèse de lésion bénigne. L´examen anatomopathologique posait le diagnostic de certitude à savoir, un épulis inflammatoire. Un contrôle post-opératoire clinique et radiologique est réalisé à quinze jours et montre une bonne cicatrisation accompagnée d´une absence de symptomatologie algique et infectieuse. Devant la présentation clinico-radiologique, les diagnostics différentiels retenus étaient le carcinome épidermoïde, une métastase de l´adénocarcinome prostatique, un épulis inflammatoire ou encore un lymphome.

**Figure 1:**
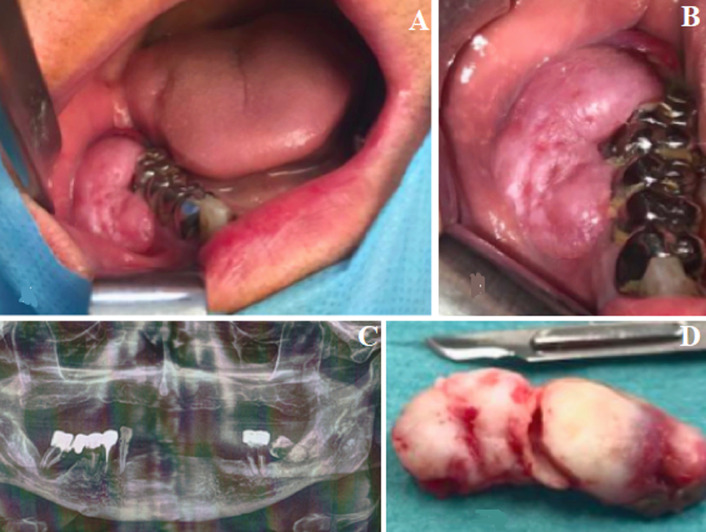
A,B) examen endobuccal initial; C) orthopantomogramme initial; D) pièce opératoire

